# Scleromyxedema in a 21 year old female patient with acute lymphoblastic leukemia: a case report

**DOI:** 10.1186/s12895-020-00118-7

**Published:** 2020-12-04

**Authors:** Hadi Rabee, Leeda Tayem, Mohammad Gharbeyah, Dina Abugaber

**Affiliations:** 1 Department of Internal Medicine , An-Najah National University Hospital , 7707, Asira Street, Nablus, Palestine; 2 Department of Dermatology , An-Najah National University Hospital , Nablus, Palestine; 3 Department of Critical Care , An-Najah National University Hospital , Nablus, Palestine

**Keywords:** Scleromyxedema, Mucinosis, Intravenous Immunoglobulin, Leukemia

## Abstract

**Background:**

Scleromyxedema is a rare, para-neoplastic, chronic, progressive condition of the Lichen myxedematosus (LM) family. The clinical picture consists of generalized confluent papular eruptions with possible systemic manifestations, which may be fatal as it still constitutes a therapeutic dilemma. Histologically, it is characterized by dermal mucin deposition, fibroblast proliferation with fibrosis, with monoclonal gammopathy in the absence of thyroid disease. Some atypical forms of the disease were reported in the literature, but none were reported in acute leukemia.

**Case presentation:**

Herein, we report a case of a 21 years old female patient, known case of acute lymphoblastic leukemia (ALL), who developed numerous hyper-pigmented erythematous papules and plaques, mainly over her thighs, lower abdomen, and sub-mammary flexures. Histopathology of skin lesions confirmed the diagnosis of atypical scleromyxedema. Her symptoms significantly improved with the use of high dose intravenous immunoglobulin (IVIG).

**Conclusions:**

Despite that scleromyxedema is associated with many hematologic disorders, it is very rarely associated with acute lymphoblastic leukemia, and a high index of suspicion is needed for diagnosis. IVIG remains a reasonable management of such a disabling disease.

## Background

Cutaneous mucinoses are a group of rare dermatological disorders, in which there is an excessive accumulation of mucin in the skin. Their clinical presentation may vary considerably making them similar to many other systemic diseases [1]. Cutaneous mucinoses are divided into 2 main broad categories: the primary mucinoses, in which the mucin deposit is the main histologic feature producing a clinically distinctive lesion, and the secondary mucinoses, where other disorders show mucin deposition as a secondary response. Diagnosis of dermal mucinoses is particularly challenging [2]. Lichen myxedematosus (LM) is an idiopathic form of primary cutaneous mucinosis. LM includes 3 clinico-pathologic subsets, which are summarized in Table 1, with their diagnostic criteria [3].

**Table 1:** Subsets of Lichen myxedematosus and their diagnostic criteria

**Generalized sclerodermoid LM “scleromyxedema”**	**Localized papular LM**	**Atypical LM**
● Generalized papular and sclerodermoid eruption. ● Mucin deposition, fibroblast proliferation, and fibrosis. ● Monoclonal gammopathy. ● Absence of thyroid disease.	● Papular or nodular plaques. ● Mucin deposition with variable fibroblast proliferation. ● Absence of both: monoclonal gammopathy and thyroid disease.	● Features that mix between the generalized and the localized forms.

In some cases, LM may be associated with certain diseases, including multiple myeloma and lymphoma [4]. It also can lead to deleterious systemic consequences, which include: dermato-neuro syndrome [5], and cardiomyopathy [6]. The treatment of these disorders is difficult and often ineffective. Many therapeutic approaches have been tried [5, 7]. However, until the date of this report, no specific approach proves to be superior to another, despite that intravenous immunoglobulin (IVIG) is becoming more utilized as a treatment.

A search of the literature found very few case reports of cutaneous mucinosis in ALL patients. Herein, we report a case of cutaneous mucinosis in ALL patient, for which IVIG was effective as a treatment.

## Case presentation

We present a case of 21 year old female patient, with unremarkable medical, family, and psychological history, who was diagnosed with ALL. The patient was on the appropriate chemotherapeutic regimen in the hematology ward. There, she developed post chemotherapy pancytopenia with neutropenic sepsis, which was treated with appropriate intravenous antibiotics (tigecycline, colistin, meropenem, and ceftazidime), and blood product transfusion as needed.

During her hospitalization, and due to the low platelet counts, the patient developed a pulmonary hemorrhage that required intubation and prolonged mechanical ventilation in the medical intensive care unit (ICU), during which her cell counts have recovered, but she remained in sepsis in the form of intractable fever with positive sputum and blood cultures for *Pseudomonas aeruginosa*.

**Fig. 1:**
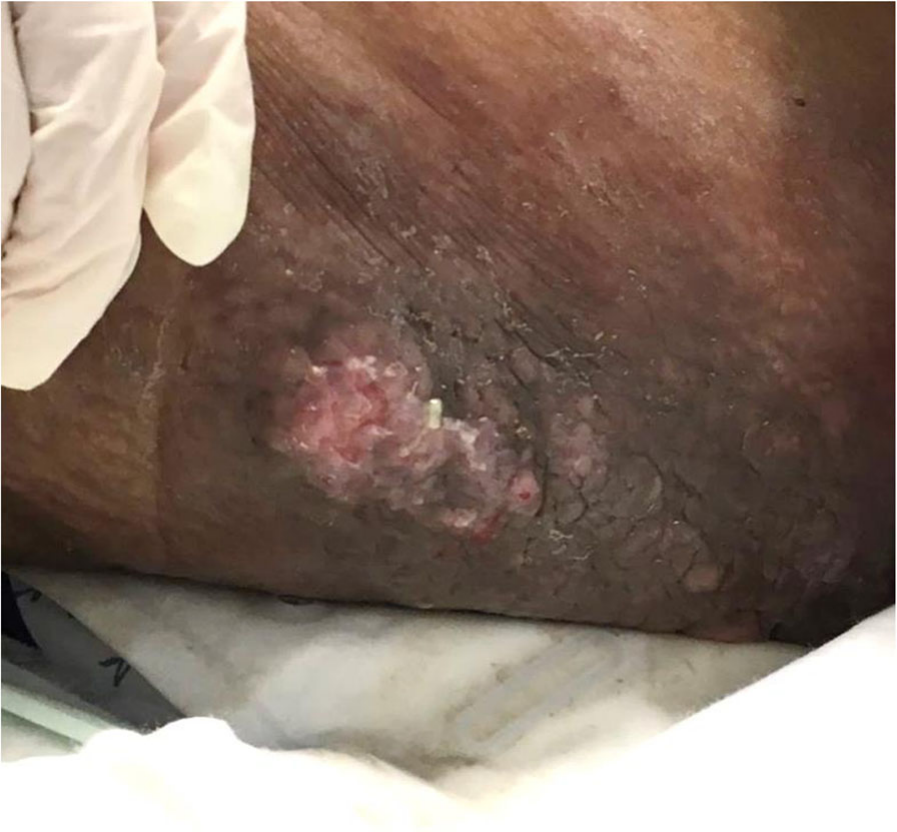
Infiltrated plaques on the thigh

During her ICU stay, patient started to develop multiple, well defined velvety, thickened, darkly pigmented papules over her thighs, external genitalia, lower abdomen, and sub-mammary flexures, which gradually enlarged, merged, and thickened to form hyper-pigmented infiltrated plaque (shown in Fig. 1). These lesions were associated with intractable fever which was not fully explained by a persistent sepsis, as the latter was well controlled with hemodynamic stability and normalization of her inflammatory markers.

Her complete blood count, routine examination and culture of urine, liver function test, renal function test, serum protein electrophoresis, blood sugar and thyroid profile were within the normal limits. Computed Tomography scan of her body during a routine evaluation of her persistent fever and sepsis showed incidental left thalamic ischemic stroke and multiple splenic infarctions. Trans-esophageal echocardiography was non contributive.

**Fig. 2:**
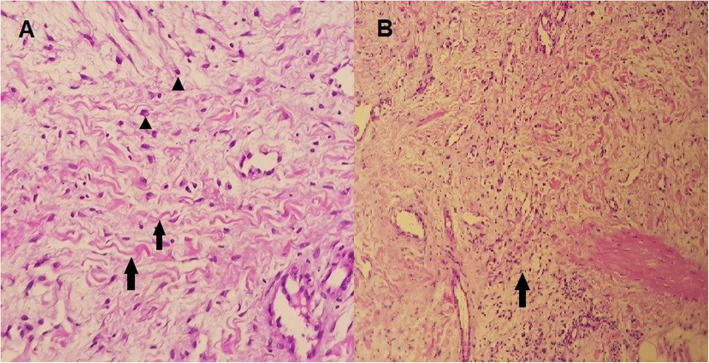
**a** Skin biopsy examination shows increased thickened collagen bundles separated by large quantities of amorphous mucinous material (*arrows*), with stellate fibroblasts (*arrow heads*) (Hematoxylin and Eosin, x40). **b** Lymphoplasmacytic infiltrate in the superficial and deep dermal blood vessels (*arrow*) (Mucicarmine, x 20)

Based on our clinical suspicion of scleromyxedema, a skin biopsy was performed and revealed abundant amounts of dermal amorphous mucinous material separating increased thickened collagen bundles, which was confirmed by mucicarmine special stain. In addition, the biopsy showed proliferation of stellate fibroblasts, and superficial and deep perivascular lympho-plasmacytic infiltrates (shown in Fig. 2).

**Fig. 3:**
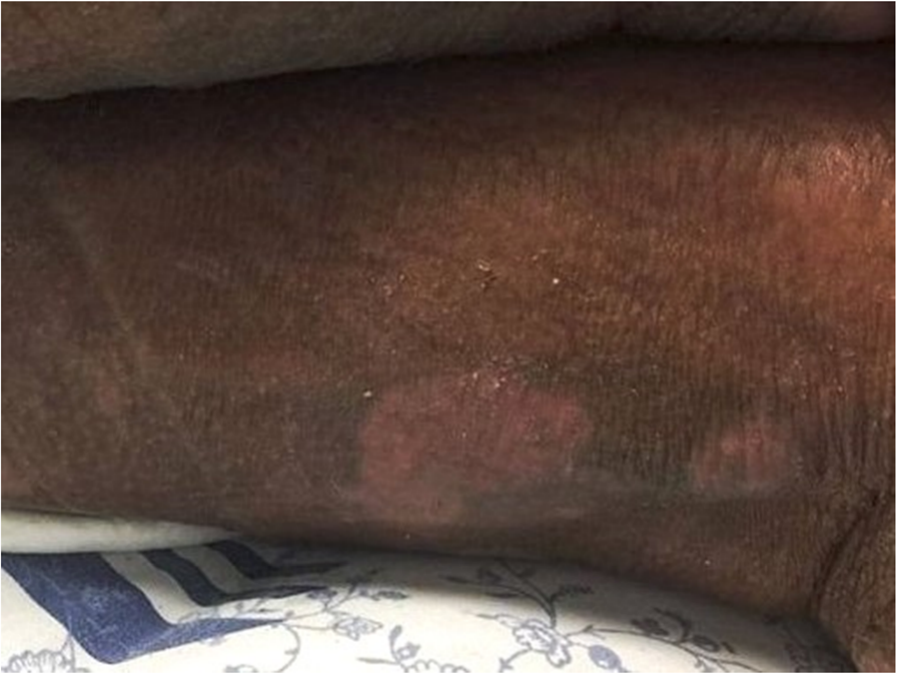
Same skin lesion two weeks after starting therapy, showing almost complete resolution with evidence of post-inflammatory hypopigmentation

The patient was started on high dose IVIG (2 g/kg) given over 2 days, in monthly cycles for a total of 6 months, and this was well tolerated. After starting the first dose of IVIG, our patient’s state considerably ameliorated in the form of improvement of cutaneous lesions (shown in Fig. 3) and complete disappearance of her intractable fever.

## Discussion and conclusion

Scleromyxedema is considered one of the rare dermatologic diseases with a chronic, progressive course of unknown etiology [8]. Scleromyxedema is considered one of the paraneoplastic dermatoses [9]. The pathogenesis of scleromyxedema is unknown, but cytokines such as interleukin-1, tumor necrosis factor-alpha, and transforming growth factor-beta, known to stimulate fibroblast proliferation in the skin, could play a role [10]. Clinical remission following stem cell transplantation suggests that the bone marrow may be a source of these circulating factors [11].

Some patients have atypical forms with features intermediate between generalized and localized LM [3]. Clinically, it is characterized by indurated erythematous, waxy papules, disseminated on the face, chest and limbs, that then coalesce to form generalized plaques causing extensive thickening and hardening of skin [6, 12]. Comparing this with our patient, we can find that she has the typical skin lesions of this disease, but a different distribution pattern notably the lack of involvement of the face and upper trunk.

As a para-neoplastic disease, different malignancies were reported in the literature in association with scleromyxedema. These included, but not limited to, monoclonal gammopathy, lymphoma, advanced gastric cancer, and very rarely, acute leukemia [6, 9]. Scleromyxedema can lead to disabling systemic manifestations, these include: dysphagia, myopathy [13], cardiomyopathy [6], central nervous system involvement, including: intractable fever, convulsion, and coma [14].

According to the clinical presentation and histopathological evidence, the diagnosis of atypical scleromyxedema was established in our patient, with systemic manifestations that might be related to her disease.

Literature review revealed no consensus regarding the therapeutic approach. Multiple regimens were used. These included the use of melphalan chemotherapy with autologous bone marrow transplant [15], and thalidomide with prednisolone [16]. High dose IVIG has recently become a more popular option and has been a first line strategy used in the recently reported cases [17]. For example, high dose IVIG for 6 months was used in a patient who was diagnosed with scleromyxedema and polyneuropathy, with improvement started after the first infusion of IVIG, and complete resolution was achieved after 3 infusions [17]. Another example of the effectiveness of IVIG was reported in 2 cases of scleromyxedema at different age groups, where skin lesions dramatically improved after the first 2 infusions of high dose IVIG (2 g/kg/month) [18]. Our case report supports the hypothesis that high dose IVIG could be adopted in treating scleromyxedema.

In conclusion, scleromyxedema is a rare but disabling skin disease. It is very rarely associated with ALL. Atypical forms require a high index of suspicion for diagnosis. A constellation of clinical and pathological features is needed for diagnosis. The management of scleromyxedema is controversial. Based on the improvement of the symptoms noted in our case, we conclude that high dose IVIG could be beneficial in treating this disease in ALL patients, supporting the recently published literature.

## Data Availability

Not applicable.

## Abbreviations

LM: Lichen myxedematosus
IVIG: Intra-venous immunoglobulin
ALL: Acute Lymphoblastic Leukemia
ICU: Intensive care unit
